# Efficacy of fine-needle aspiration cytology for a thyroid abscess in children: Two case reports

**DOI:** 10.3892/etm.2015.2189

**Published:** 2015-01-19

**Authors:** YAPING LU, JIE ZHANG, XIAOYU LIANG, MENG HU, RONGXIU ZHENG, LIQIN LI

**Affiliations:** 1 Department of Ultrasound, Tianjin Chest Hospital, Tianjin 300051, P.R. China; 2 Department of General Surgery, General Hospital, Tianjin Medical University, Tianjin 300052, P.R. China; 3 Graduate School, Tianjin Medical University, Tianjin 300070, P.R. China; 4 Department of Pediatrics, General Hospital, Tianjin Medical University, Tianjin 300052, P.R. China

**Keywords:** acute suppurative thyroiditis, thyroid abscess, ultrasound-guided fine-needle aspiration cytology, children

## Abstract

A thyroid abscess caused by acute suppurative thyroiditis (AST) is a rare form of thyroid nodule, and is most common in children, particularly in the first decade of life. The echotexture of an abscess may vary depending on the extent of internal debris or hemorrhage and on the peripheral and interval vascular flow; thus, a definitive diagnosis of AST is difficult to establish. The present study reports two cases of a thyroid abscess in children caused by viridans streptococci, diagnosed using ultrasound-guided fine-needle aspiration cytology (FNAC) and bacterial culturing. FNAC of the thyroid gland may be used extensively in children for the diagnosis of AST and thyroid abscesses. In addition, FNAC is an efficient method for differentiating between benign and malignant nodules of the thyroid gland, in order to ensure that the appropriate treatment is administered.

## Introduction

Acute suppurative thyroiditis (AST), an inflammatory disease of the thyroid gland, is extremely rare in adults, since they typically exhibit a strong resistance to local infection (1). Thyroid abscesses are more likely to occur in children in instances of congenital anomaly (2). The majority of AST cases are caused by Gram-positive *Streptococcus* species (3). If AST is left untreated, it may enter the neck or extend to the chest. Rupture of the abscess into the esophagus or trachea is also possible. Despite outpatient parenteral antibiotic therapy, a number of cases have required surgical drainage in conjunction with the removal of any associated anatomic abnormality, in order to decrease the recurrence of abscess formation (4). Abscesses may exhibit anechoic or internal echoes; therefore, it may not be possible to establish a definitive diagnosis using standard ultrasonography alone. In such cases, ultrasound-guided fine-needle aspiration cytology (FNAC) and bacterial culture may be performed to confirm the diagnosis. The present study reports two cases of AST that were accompanied by abscesses, together with a review of the literature.

## Case reports

### Case 1

**Figure 1:**
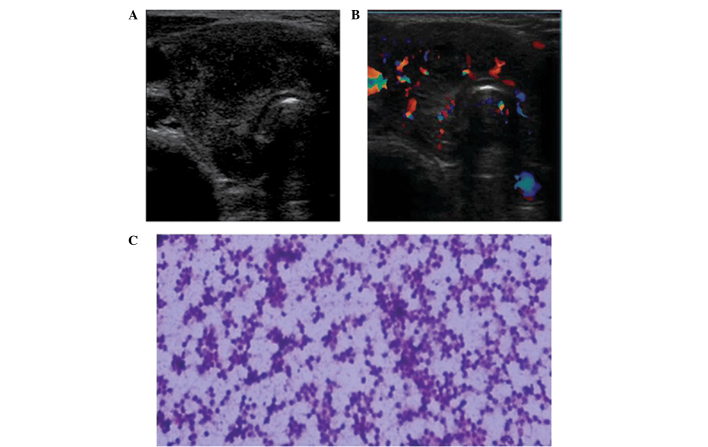
Case 1. (A) Thyroid ultrasonography showing a 30×25×21 mm^3^ heterogeneous hypoechoic mass with hyperechoic spots. (B) Thyroid ultrasonography showing the mass with interval vascular flow. (C) Cytological findings of the fine-needle aspiration revealed no follicular cells, only a mixture of various inflammatory cells (hematoxylin-eosin stain; magnification, ×200).

A seven-year-old male patient presented with a painful swelling in the left side of the neck, a sore throat and a hoarse voice for one day, as well as fever for four days. Laboratory analyses revealed a leukocyte count of 12,940/ml, with 66.1% neutrophils and 26% lymphocytes, and normal thyroid function. The blood culture was sterile with no bacterial presence; however, the patient received ampicillin on admission. Ultrasonography examination of the neck revealed a 30×25×21 mm^3^ heterogeneous hypoechoic mass with hyperechoic spots, which almost filled the entire left lobe and isthmus of the thyroid gland. In addition, peripheral and interval vascular flow was observed, indicating a malignant nodule (Fig. 1A and B). A FNAC procedure was performed using a disposable 5-ml syringe on the sixth day following patient admission. The aspiration fluid was expressed onto slides, and smears were prepared. One of the smears was immediately fixed in 95% ethyl alcohol for subsequent hematoxylin-eosin staining. The remaining smears were air-dried and stained rapidly with Diff-Quik (Baso diagnostics Inc., Zhuhai, China). The FNAC procedure revealed no evidence of nodule malignancy. A mixture of various inflammatory cells were observed; however, no follicular cells were identified (Fig. 1C). A 0.1-ml sample of pus-like fluid was extracted from the abscess by aspiration, which subsequently grew colonies of viridans streptococci when cultured, confirming the diagnosis of AST. However, the abscess was not surgically drained as the patient exhibited clinical improvement after receiving antibiotics. Written informed patient consent was obtained from the patient’s family prior to participation in the present study.

### Case 2

**Figure 2:**
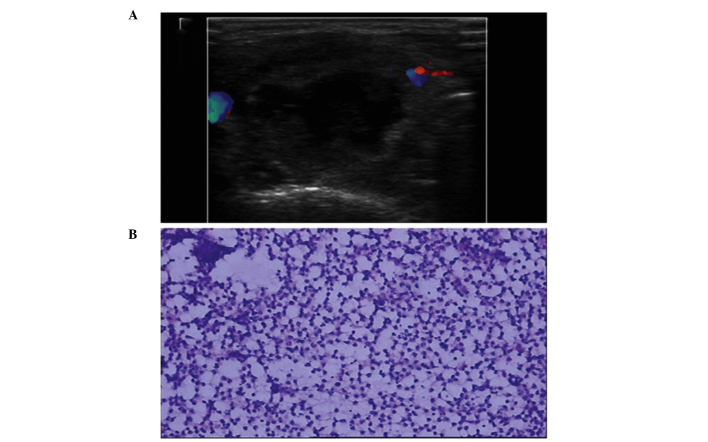
Case 2. (A) Thyroid ultrasonography showing a 23×21×20 mm^3^ cystic mixed mass without interval vascular flow. (B) Cytological findings of the fine-needle aspiration revealed numerous neutrophils and a number of macrophages (hematoxylin-eosin stain; magnification, ×200).

A nine-year-old male patient was admitted to the General Hospital (Tianjin, China) suffering from painful swelling and fever for two days. Laboratory examinations revealed a leukocyte count of 12,400/ml, consisting of 76.4% neutrophils and 18.2% lymphocytes, and normal thyroid function. A 23×21×20 mm^3^ cystic mixed mass was observed in the left lobe of thyroid gland, with no interval vascular flow (Fig. 2A). A FNAC examination was subsequently performed. A 2-ml sample of purulent yellow-brown fluid was extracted from the abscess, after which the anechoic area disappeared. A number of the smears were rapidly stained with Diff-Quik and the remainder were stained by the conventional hematoxylin-eosin method. Aspiration cytology revealed no thyroid follicular cells; however, numerous neutrophils and a number of macrophages were observed (Fig. 2B). Culture of the aspirated fluid resulted in the growth of viridans streptococci. Thus, a diagnosis of AST with an abscess was confirmed. The patient was treated with ampicillin upon admission and the fever subsided two days after the FNAC procedure. Written informed patient consent was obtained from the patient’s family prior to participation in the present study.

## Discussion

The thyroid gland is known to strongly resist infections due to its encapsulation, abundant blood supply and lymph nodes, iodine concentration and generation of hydrogen peroxide (5). A thyroid abscess caused by AST is an extremely rare form of solitary thyroid nodule, which results from an infection via a pyriform sinus fistula (6) and is most commonly observed in children (7). According to the results of previously published studies and the anatomical and histological results of the current case reports, the authors of the present study believe that the development of the fistulas originated from the embryonic remnant of the fourth branchial pouch, which regularly presents in the first decade of life (8–10).

Streptococcal, staphylococcal and pneumococcal are the three types of infection most commonly associated with cases of AST (3). The disorder is primarily characterized by a sudden enlargement and tenderness of the thyroid gland, accompanied by a fever, sore throat, dysphagia, hoarseness and a limited range of head movement (11). Ultrasound examination provides real-time imaging of the affected area without the use of ionizing radiation, and is able to reveal intra- or extra-thyroid abscesses and solid or mixed lesions of the thyroid gland. However, the echotexture of an abscess may vary due to extensive internal debris or hemorrhage, accompanied by peripheral and interval vascular flow. In such cases, a definitive diagnosis may be impossible to establish using ultrasonography alone, and FNAC may play a significant role in discriminating between benign and malignant lesions, while generating few complications (12). The use of a rapid staining protocol allows the quality of the stain, smear and specimen to be assessed immediately (13). In the two present cases, a diagnosis of AST was rapidly and easily established on the basis of Diff-Quik staining only. However, additional materials may be evaluated using hematoxylin-eosin staining to aid a more specific final diagnosis. If there is a notable anechoic area in the lesion, pus-fluid can be collected during the FNAC procedure and cultured. In the present cases, the cultures revealed the presence of veridians streptococci, which are normal microbiota encountered in the respiratory tract. The existence of a pyriform sinus fistula was suspected, where the sinus exhibits a proximal opening in the apex of the pyriform fossa and the distal end extends to the thyroid, resulting in the thyroid becoming prone to inflammation and infection.

In the present cases, the viridans streptococci responded to treatment with ampicillin; thus, surgical drainage of the abscesses was unnecessary. However, AST patients require a close clinical follow-up to ensure the complete elimination of the infection. In a limited number of cases, surgical excision of the fistula may be performed in combination with a direct laryngoscopy examination, in order to prevent the recurrence of thyroiditis. In case 1, ultrasonography revealed a heterogeneous hypoechoic mass with hyperechoic spots, similar to a neoplasm, which may be falsely diagnosed as a thyroid malignancy. A combination of FNAC and bacterial culture may aid the diagnosis of AST. Furthermore, during the aspiration procedure, abscess fluids may be collected, and further surgical drainage is rarely required, as was observed in case 2. In cases exhibiting a large abscess, surgical drainage should be followed by antibiotic treatment.

In summary, FNAC of the thyroid gland is useful for establishing a diagnosis of AST with a thyroid abscess. This procedure may prove to be a less invasive and more efficient, timely and feasible method for discriminating between benign and malignant thyroid gland nodules, in order to ensure that the appropriate treatment is administered.

## References

[1] Paes JE, Burman KD, Cohen J (2010). Acute bacterial suppurative thyroiditis: a clinical review and expert opinion. Thyroid.

[2] Pal I, Sengupta S, Balakrishnan R, Gupta A (2009). Fourth branchial pouch sinus - an unusually late initial presentation. Indian J Otolaryngol Head Neck Surg.

[3] Bravo E, Grayev A (2011). Thyroid abscess as a complication of bacterial throat infection. J Radiol Case Rep.

[4] Parida PK, Gopalakrishnan S, Saxena SK (2014). Pediatric recurrent acute suppurative thyroiditis of third branchial arch origin - our experience in 17 cases. Int J Pediatr Otorhinolaryngol.

[5] Hong JT, Lee JH, Kim SH (2013). Case of concurrent Riedel’s thyroiditis, acute suppurative thyroiditis, and micropapillary carcinoma. Korean J Intern Med.

[6] Prajapaty B, Shah B (1997). Recurrent cervical abscess due to pyriform sinus fistula - a case report. Indian J Otolaryngol Head Neck Surg.

[7] Pearce EN, Farwell AP, Braverman LE (2003). Thyroiditis. N Engl J Med.

[8] Desai AA, Pandya VK, Chougule S, Nair U (2006). Recurrent thyroid abscess - Is it a fourth branchial archanomaly?. Indian J Otolaryngol Head Neck Surg.

[9] Nicoucar K, Giger R, Pope HG, Jaecklin T, Dulguerov P (2009). Management of congenital fourth branchial arch anomalies: a review and analysis of published cases. J Pediatr Surg.

[10] Shrime M, Kacker A, Bent J, Ward RF (2003). Fourth branchial complex anomalies: a case series. Int J Pediatr Otorhinolaryngol.

[11] Ghaemi N, Sayedi J, Bagheri S (2014). Acute suppurative thyroiditis with thyroid abscess: a case report and review of the literature. Iran J Otorhinolaryngol.

[12] Vasudev V, Hemalatha AL, Rakhi B, Githanjali S (2014). Efficacy and pitfalls of FNAC of thyroid lesions in children and adolescents. J Clin Diagn Res.

[13] Powers CN (1998). Diagnosis of infectious diseases: a cytopathologist’s perspective. Clin Microbiol Rev.

